# Validation of genome-wide associated variants for Kawasaki disease in a Taiwanese case–control sample

**DOI:** 10.1038/s41598-020-68673-0

**Published:** 2020-07-16

**Authors:** Ming-Ren Chen, Tzu-Yang Chang, Nan-Chang Chiu, Hsin Chi, Kuender D. Yang, Lung Chang, Daniel Tsung-Ning Huang, Fu-Yuan Huang, Ya-Ping Lien, Wen-Shan Lin, Chiung-Ling Lin, Luan-Yin Chang, Yann-Jinn Lee

**Affiliations:** 1 Department of Pediatric Cardiology, MacKay Children’s Hospital, Taipei, Taiwan; 2 Department of Medical Research, MacKay Memorial Hospital, No. 45, Min-Sheng Road, Tamshui District, New Taipei City, 25160 Taiwan; 3 Department of Nursing, MacKay Junior College of Medicine, Nursing, and Management, Taipei, Taiwan; 4 Department of Medicine, Mackay Medical College, New Taipei City, Taiwan; 5 Department of Pediatric Infectious Diseases, MacKay Children’s Hospital, Taipei, Taiwan; 6 Department of Pediatric Allergy and Immunology, MacKay Children’s Hospital, Taipei, Taiwan; 7 Department of Pediatrics, College of Medicine, National Taiwan University, Taipei, Taiwan; 8 Department of Pediatrics, National Taiwan University Children’s Hospital, No. 8, Zhongshan S. Rd, Zhongzheng District, Taipei, 100 Taiwan; 9 Institute of Biomedical Sciences, Mackay Medical College, New Taipei, Taiwan; 10 Department of Pediatric Endocrinology, MacKay Children’s Hospital, Taipei, Taiwan; 11 Department of Pediatrics, School of Medicine, College of Medicine, Taipei Medical University, Taipei, Taiwan; 12 Department of Pediatrics, MacKay Memorial Hospital, No. 45, Min-Sheng Road, Tamshui District, New Taipei City, 25160 Taiwan

**Keywords:** Genetics, Immunology, Biomarkers, Diseases, Medical research, Molecular medicine, Pathogenesis, Risk factors

## Abstract

Kawasaki disease (KD) is an acute febrile systemic vasculitis of unknown etiology that affects infants and young children. Considerable evidence supports the hypothesis that there is a genetic basis for KD susceptibility. Genome-wide association studies (GWAS) have identified several genetic variants associated with KD. This study aims to replicate three novel KD-associated single nucleotide polymorphisms (SNPs), identified by GWAS in Japanese, in a Taiwanese population. Associations between these SNPs and development of coronary artery lesions (CALs) were also investigated. The *rs2254546 A/G*, *rs2857151 A/G*, and *rs4813003 C/T* SNPs were genotyped in 681 children with KD and 563 ethnically-matched healthy controls using TaqMan Assay or DNA sequencing. We found *rs2254546* and *rs4813003* SNPs were significantly associated with KD (*G* allele, odds ratio [OR] = 1.54, *P* = 1.0 × 10^–5^; *C* allele, OR = 1.32, *P* = 8.1 × 10^–4^). However, no evidence for associations with CAL development was observed. Our study successfully validates associations of the *rs2254546* and *rs4813003* SNPs with KD in a Taiwanese population. Further functional studies of the SNPs are important in understanding the pathogenesis of KD.

## Introduction

Kawasaki disease (KD; OMIM 611775) is an acute febrile vasculitis syndrome that occurs predominantly in children under 5 years of age^1^. The major clinical symptoms of KD are fever lasting for longer than 5 days, bilateral non-purulent conjunctivitis, cervical lymphadenopathy, erythema of the palms and soles, diffuse mucosal inflammation, and polymorphous skin rashes^2,3^. If left untreated, around 15–25% children with KD develop coronary artery lesions (CALs), making this disease the most important cause of acquired heart disease in children of developed countries^4^. Although its etiology remains unclear, higher incidence rates in east Asian populations and higher risk in siblings imply that genetic factors play important roles in the development of KD^5,6^.

Genome-wide association study (GWAS) is a systematic approach to analyze the correlation between genetic markers and human diseases. This method allows one to find out disease-associated variants across the genome in large population groups. GWAS has been successfully undertaken for different kinds of complex genetic diseases and discovers a large number of associated loci. As of 2018, the GWAS catalog contains over 5,600 studies and 71,673 variant-trait associations from 3,567 publications^7^. KD, a complex disorder, has also been studied by GWAS to seek potentially associated variants. Until now, a total of 7 KD GWAS have been conducted in different races and some novel associated KD loci are identified^8–14^.

Because of difference in genetic architectures based on ethnicity, replications of GWAS findings in other racial groups are needed to verify the susceptible genes for KD. Therefore, we aim to determine whether GWAS-identified single nucleotide polymorphisms (SNPs) can be replicated in a Taiwanese population by using a case–control study design with 681 children with KD and 563 healthy controls. In addition, we examined associations between the GWAS-significant SNPs and CAL development in children with KD.

## Results

### Association between SNPs and KD susceptibility

The genotyping of SNPs *rs2254546*, *rs2857151*, and *rs4813003* was successful in 563 controls and 681 children with KD (Tables 1, 2, 3). The observed genotype frequencies for these polymorphisms were in agreement with Hardy–Weinberg equilibrium in the controls. Association tests revealed significant differences in the distribution of genotypes and alleles of SNPs *rs2254546 A/G* (*P* = 3.5 × 10^–5^ and 1.0 × 10^–5^) and *rs4813003 C/T* (*P* = 2.0 × 10^–3^ and 8.1 × 10^–4^) between controls and KD children, which remained significant after Bonferroni correction (*P*_*c*_ < 0.05) (Tables 1, 3). The frequencies of *rs2254546 G/G* genotype and *G* allele (OR 1.69, 95% CI 1.34–2.13; OR 1.54, 95% CI 1.27–1.86) and *rs4813003 C/C* genotype and *C* allele (OR 1.50, 95% CI 1.18–1.90; OR 1.32, 95% CI 1.12–1.54) significantly increased in children with KD than in the controls. The genotype and allele frequencies for SNP *rs2857151 A/G* did not differ significantly (*P* = 0.81 and 0.69) (Table 2).

**Table 1:** Genotype and allele frequencies of the *rs2254546 A/G* polymorphism in controls and KD children, and in KD children with and without CAL.

		Genotypes, n (%)				Alleles, n (%)	
		AA	AG	GG		A	G
Control (n = 563)		34 (6.0)	223 (39.6)	306 (54.4)		291 (25.8)	835 (74.2)
KD (n = 681)		26 (3.8)	200 (29.4)	455 (66.8)		252 (18.5)	1,110 (81.5)
*P* value	3.5 × 10^–5^				1.0 × 10^–5^		
OR (95% CI)		0.62 (0.37–1.04)	0.63 (0.50–0.80)	1.69 (1.34–2.13)		0.65 (0.54–0.79)	1.54 (1.27–1.86)
KD without CAL (n = 524)		19 (3.6)	153 (29.2)	352 (67.2)		191 (18.2)	857 (81.8)
KD with CAL (n = 157)		7 (4.5)	47 (29.9)	103 (65.6)		61 (19.4)	253 (80.6)
*P* value	0.87				0.63		
OR (95% CI)		1.24 (0.51–3.01)	1.04 (0.70–1.53)	0.93 (0.64–1.36)		1.08 (0.79–1.49)	0.92 (0.67–1.27)

*KD* Kawasaki disease, *CAL* coronary artery lesion, *OR* odds ratio, *CI* confidence interval.

**Table 2:** Genotype and allele frequencies of the *rs2857151 A/G* polymorphism in controls and KD children, and in KD children with and without CAL.

		Genotypes, n (%)				Alleles, n (%)	
		AA	AG	GG		A	G
Control (n = 563)		40 (7.1)	218 (38.7)	305 (54.2)		298 (26.5)	828 (73.5)
KD (n = 681)		42 (6.2)	267 (39.2)	372 (54.6)		351 (25.8)	1,011 (74.2)
*P* value	0.81				0.69		
OR (95% CI)		0.86 (0.55–1.35)	1.02 (0.81–1.28)	1.02 (0.81–1.27)		0.96 (0.81–1.15)	1.04 (0.87–1.24)
KD without CAL (n = 524)		33 (6.3)	197 (37.6)	294 (56.1)		263 (25.1)	785 (74.9)
KD with CAL (n = 157)		10 (6.4)	69 (43.9)	78 (49.7)		89 (28.3)	225 (71.7)
*P* value	0.34				0.25		
OR (95% CI)		1.01 (0.49–2.10)	1.31 (0.91–1.87)	0.77 (0.54–1.12)		1.18 (0.89–1.57)	0.85 (0.64–1.12)

*KD* Kawasaki disease, *CAL* coronary artery lesion, *OR* odds ratio, *CI* confidence interval.

**Table 3:** Genotype and allele frequencies of the *rs4813003 C/T* polymorphism in controls and KD children, and in KD children with and without CAL.

		Genotypes, n (%)				Alleles, n (%)	
		CC	CT	TT		C	T
Control (n = 563)		168 (29.8)	287 (51.0)	108 (19.2)		623 (55.3)	503 (44.7)
KD (n = 681)		265 (38.9)	314 (46.1)	102 (15.0)		844 (62.0)	518 (38.0)
*P* value	2.0 × 10^–3^				8.1 × 10^–4^		
OR (95% CI)		1.50 (1.18–1.90)	0.82 (0.66–1.03)	0.74 (0.55–1.00)		1.32 (1.12–1.54)	0.76 (0.65–0.89)
KD without CAL (n = 524)		206 (39.3)	243 (46.4)	75 (14.3)		655 (62.5)	393 (37.5)
KD with CAL (n = 157)		59 (37.6)	71 (45.2)	27 (17.2)		189 (60.2)	125 (39.8)
*P* value	0.67				0.46		
OR (95% CI)		0.93 (0.64–1.34)	0.95 (0.67–1.37)	1.24 (0.77–2.01)		0.91 (0.70–1.17)	1.10 (0.85–1.43)

*KD* Kawasaki disease, *CAL* coronary artery lesion, *OR* odds ratio, *CI* confidence interval.

### Association between SNPs and CAL development

Based on the CAL data of children with KD, we further examined the association between investigated polymorphisms and CAL formation. No statistically significant differences were observed in genotype and allele frequencies of any of the 3 SNPs between children with CAL and those without CAL (*P* = 0.87 and 0.63 for *rs2254546*, *P* = 0.34 and 0.25 for *rs2857151*, *P* = 0.67 and 0.46 for *rs4813003*) (Tables 1, 2, 3).

## Discussion

Replication of positive findings from genetic association studies in independent populations has become the gold standard to validate susceptibility genes. In this study, we attempted to replicate 3 GWAS-identified SNPs for Japanese children with KD in a Taiwanese population. We found that *rs2254546 G/G* genotype and *G* allele and *rs4813003 C/C* genotype and *C* allele were associated with increased risk of KD. These findings validated *rs2254546 G* allele and *rs4813003 C* allele confer risk to KD in our study population, which was consistent with the Japanese GWAS^12^. However, the association between *rs2857151 G* allele and KD cannot be replicated in our study. When the analysis was restricted to the CAL outcome, we found that none of these replicated SNPs was risk factor for CAL development in Taiwanese children with KD.

The advent of GWAS has made it a reality to study the genetic mechanisms underlie KD pathogenesis in an unbiased and efficient way. The first GWAS of KD performed in a Caucasian population found that *rs17531088* (*EXOC1*) and *rs7199343* (*ZFHX3*) were the most significantly associated SNPs^8^. A Korean GWAS reported by Kim et al*.* revealed that KD was associated with *rs527409* (*1p31*)^9^. A GWAS conducted in Taiwanese population showed that 10 SNPs located in 3 novel loci (*COPB2*, *ERAP1*, and *IGHV*) were associated with KD^10^. Khor et al*.* demonstrated that significant associations with KD at the GWAS level were observed in *rs1801274* (*FCGR2A*), *rs2233152* (near *MIA* and *RAB4B*), and *rs28493229* (*ITPKC*) in a combined European and Asian population^11^. Interestingly, a Japanese GWAS and a Taiwanese GWAS published concurrently in the same journal reported *BLK*, *CD40*, and *HLA* as susceptibility loci for KD^12,13^. The most recent GWAS in Korean population discovered *NMNAT2*, *BLK*, and *HCP5* loci contributed to KD risk^14^.

SNP *rs2254546* is located between *FAM167A* and *BLK* gene loci, a region known to have genetic associations with autoimmune diseases like systemic lupus erythematosus^15–17^, rheumatoid arthritis^18,19^, and systemic sclerosis^20,21^. *BLK* encodes B lymphoid kinase (Blk), a member of the Src family of kinases, which mediates downstream signaling of B cell receptors and affects the proliferation, differentiation and tolerance of B cells^22^. It has also been found that Blk is required to regulate T-cell mediated proinflammatory cytokine production^23^. *FAM167A* gene was recently reported to contribute to immune function including antibody isotype determination and immunoglobulin production^24^. In addition to our current study, genetic associations of *rs2254546* with KD have been validated in Chinese population^25,26^. Together, these findings support both humoral and cellular immunity may be involved in KD pathogenesis.

*rs4813003*, located 4.9 kb downstream of *CD40* gene, was also replicated successfully in our study population. CD40 is a cell surface receptor that belongs to the tumor necrosis factor receptor superfamily and has been found expressed on antigen-presenting cells such as B cells, macrophages, and dendritic cells^27^. The dyadic interaction of CD40 and CD40 ligand plays an essential role for proliferation, differentiation, and activation of B and T cells^28,29^. Besides, CD40 has been proposed as a contributing factor for immune-mediated inflammatory diseases such as systemic lupus erythematosus^30^, rheumatoid arthritis^31^, Crohn’s disease^32^, Graves’ disease^33^, and psoriasis^34^. Replication of the *rs4813003* SNP in Chinese population also revealed it was nominally associated with KD susceptibility^35^. It is therefore conceivable to infer that CD40 is prominent in determining the risk for KD.

In summary, our study confirmed the associations of *rs2254546* and *rs4813003* polymorphisms with KD susceptibility in a Taiwanese population but these SNPs did not contribute to the development of clinically evident CAL. It will be necessary to validate or replicate these associations in other independent large-scale cohorts of different ethnicities.

## Methods

### Study population

The case group contained 681 unrelated Taiwanese children with KD (423 boys, 258 girls; mean age 1.8 years, range 0.1–8.3 years), which were consecutively recruited from MacKay Memorial Hospital in Taiwan between 2005 and 2019. A portion of the KD cohort has ever been included in our previous studies^36–39^. Diagnosis of KD was made according to the diagnostic criteria of the American Heart Association^2^. Oral aspirin and intravenous gamma-globulin therapy were prescribed as soon as the diagnosis was made. All children were examined by 2-dimensional echocardiography during the febrile stage and after hospital discharge. CALs were defined by an internal arterial diameter was ≥ 3 mm in children < 5 years old, ≥ 4 mm in children ≥ 5 years old, or > 1.5 times that of an adjacent artery^40^. Of all patients studied, 157 developed CAL while the remaining revealed no evidence of CAL.

The control group consisted of 563 unrelated ethnic-matched healthy people (223 males, 340 females; mean age 35.8 years, range 10.1–66.4 years) without a history of KD, autoimmune, or allergic disease. The Institutional Review Board of MacKay Memorial Hospital approved this study and written informed consent was obtained from either the participants or their parents/guardians. All procedures of this study conformed to the principles of the Declaration of Helsinki. All methods were conducted in accordance with the relevant guidelines and regulations.

### SNP selection and analysis

Inclusion criteria of the candidate SNPs was set at genome-wide significance of combined *P* value less than 5.0 × 10^–8^ and was not previously validated in our KD patients. Based on these conditions, a total of three polymorphisms identified in the Japanese GWAS^12^ were chosen for replication: *rs2254546* at 8p22-23 (*FAM167A-BLK*), *rs2857151* at 6p21.3 (*HLA-DQB2–DOB*), and *rs4813003* at 20q13 (*CD40*). Genomic DNA was extracted from peripheral blood sample of each participant using the QIAamp DNA Blood Mini Kit (Qiagen, Hilden, Germany). The *rs2254546* and *rs4813003* SNPs were genotyped by the TaqMan Allelic Discrimination Assay (Applied Biosystems, Foster City, CA) as previously described^41^.

Genotyping for the *rs2857151* polymorphism was analyzed by PCR amplification followed by DNA sequencing. The primer pairs used were 5′-CCT ATC ATT TTG TGG GAG GTA GT-3′ (forward) and 5′-CCC AGC TGT ACC TGC CTT AG-3′ (reverse). PCR was performed at a 50 μL reaction volume containing 100 ng of genomic DNA, 0.2 μM of each primer, and 25 μL of 2 × EmeraldAmp MAX PCR Master Mix (Takara Biotechnology, Dalian City, Liaoning, China). PCR cycling parameters were: one cycle of 98 °C for 1 min, 25 cycles of 98 °C for 10 s, 49 °C for 10 s and 72 °C for 30 s, followed by one cycle of 72 °C for 5 min. The PCR fragments were sequenced on an ABI PRISM 3700 Automated Sequencer (Applied Biosystems) using the PCR primers.

### Statistical analysis

The Hardy–Weinberg equilibrium for genotypes in controls and genotype and allele frequencies associated with the KD susceptibility and CAL formation were assessed by the χ^2^ test. Odds ratios and 95% confidence intervals were also calculated. The Bonferroni correction was used to calculate corrected *P* (*P*_*c*_) values. Two-tailed *P*_*c*_ values of less than 0.05 were considered to be statistically significant. Prior to the study, statistical power to detect effects of these SNPs on KD susceptibility was calculated using the Quanto Ver. 1.1 software (Department of Preventive Medicine, University of Southern California, CA, USA). The study was designed to have a power of over 98% at a 5% significance level to determine a relative risk of 1.5 conferred by the risk genotype of each SNP with the KD prevalence of 65.3 per 10,000 children^42^.
